# Impact of a computerized decision support tool deployed in two intensive care units on acute kidney injury progression and guideline compliance: a prospective observational study

**DOI:** 10.1186/s13054-020-03343-1

**Published:** 2020-11-23

**Authors:** Christopher Bourdeaux, Erina Ghosh, Louis Atallah, Krishnamoorthy Palanisamy, Payaal Patel, Matthew Thomas, Timothy Gould, John Warburton, Jon Rivers, John Hadfield

**Affiliations:** 1 Bristol Royal Infirmary, Anesthesia, University Hospital Bristol, Bristol, UK; 2 Philips Research North America, 222 Jacobs Street, Cambridge, MA 02141 USA; 3 Philips Innovation Campus, Bangalore, India

**Keywords:** Acute kidney injury, Intensive care, Early detection, AKI management

## Abstract

**Background:**

Acute kidney injury (AKI) affects a large proportion of the critically ill and is associated with worse patient outcomes. Early identification of AKI can lead to earlier initiation of supportive therapy and better management. In this study, we evaluate the impact of computerized AKI decision support tool integrated with the critical care clinical information system (CCIS) on patient outcomes. Specifically, we hypothesize that integration of AKI guidelines into CCIS will decrease the proportion of patients with Stage 1 AKI deteriorating into higher stages of AKI.

**Methods:**

The study was conducted in two intensive care units (ICUs) at University Hospitals Bristol, UK, in a before (control) and after (intervention) format. The intervention consisted of the AKIN guidelines and AKI care bundle which included guidance for medication usage, AKI advisory and dashboard with AKI score. Clinical data and patient outcomes were collected from all patients admitted to the units. AKI stage was calculated using the Acute Kidney Injury Network (AKIN) guidelines. Maximum AKI stage per admission, change in AKI stage and other metrics were calculated for the cohort. Adherence to eGFR-based enoxaparin dosing guidelines was evaluated as a proxy for clinician awareness of AKI.

**Results:**

Each phase of the study lasted a year, and a total of 5044 admissions were included for analysis with equal numbers of patients for the control and intervention stages. The proportion of patients worsening from Stage 1 AKI decreased from 42% (control) to 33.5% (intervention), *p* = 0.002. The proportion of incorrect enoxaparin doses decreased from 1.72% (control) to 0.6% (intervention), *p* < 0.001. The prevalence of any AKI decreased from 43.1% (control) to 37.5% (intervention), *p* < 0.05.

**Conclusions:**

This observational study demonstrated a significant reduction in AKI progression from Stage 1 and a reduction in overall development of AKI. In addition, a reduction in incorrect enoxaparin dosing was also observed, indicating increased clinical awareness. This study demonstrates that AKI guidelines coupled with a newly designed AKI care bundle integrated into CCIS can impact patient outcomes positively.
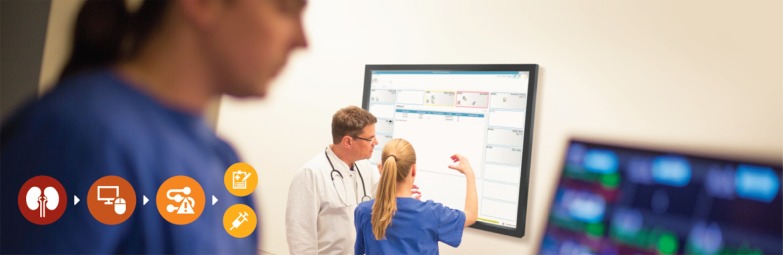

## Background

Acute kidney injury (AKI) is a common occurrence in the critically ill, developing in more than 50% of patients at some point during a critical care admission [1]. It increases mortality, length of stay and healthcare cost [2]. Any strategy which reduces the incidence of AKI has the potential to improve outcome, reduce cost and optimize resource utilization. Despite clear guidelines on the recognition and management of AKI [3], several reports have highlighted a need to improve the application of these guidelines in routine care [4, 5].

The critical care management of AKI consists of supportive therapy and minimizing further kidney injury by treating the underlying cause, avoiding nephrotoxic drugs, optimizing fluid status and perfusion pressure and the use of renal replacement therapy. However, there is no single remedy for established AKI [6] and early identification of patients with AKI has the potential to improve outcomes [5]. Automated electronic alerts may be expected to improve early detection of AKI, while clinical decision support systems (CDSS) might further support clinicians in diagnosis, drug dosing and management. Previous studies applying these approaches to AKI have shown variability in results [7–10]. This is likely due to population differences as well as differences in the nature of the alert and action prompted.

Critical care clinical information systems (CCIS) are increasingly deployed to display large data sets to frontline clinicians. The format of these data is often not optimized to enable evidence-based decision making at the point of care. We have previously shown that simple changes to the structure and display of data within a CCIS can significantly impact decision making in prescribing and ventilation practice [11]. In this study, we evaluate the impact of a previously validated algorithm [12] when embedded within a newly designed workflow as part of a CCIS in 2 separate intensive care units in a large UK teaching hospital.

In particular, we assessed the impact of an electronic AKI alert embedded within the existing electronic patient record on the progression of AKI in critically ill patients. Our hypothesis is that an AKI CDSS combined with a care bundle will improve patient outcomes as defined by a reduction in proportion of patients with Stage 1 AKI developing higher stages of AKI. In order to understand the impact of the CDSS on clinician behavior, we also evaluated the effect of the alert on a proxy for guideline compliance by examining the dosing of a commonly prescribed drug, enoxaparin, that requires adjustment in the context of AKI.

## Methods

### Setting and enrollment

The study was conducted in two ICUs at University Hospitals Bristol, UK, a closed format tertiary medical and surgical ICU (GICU) and closed format tertiary cardiac ICU (CICU). GICU admits over 1200 patients per year and CICU is a regional cardiac surgical center with over 1800 admissions a year. Both units have been using the Philips IntelliSpace Critical Care and Anesthesia (ICCA) system since 2015. This system automatically collects all information relating to patient care including laboratory data and hourly vitals and observations. It also allows the development and deployment of clinical decision support software and analysis tools.

**Fig. 1:**
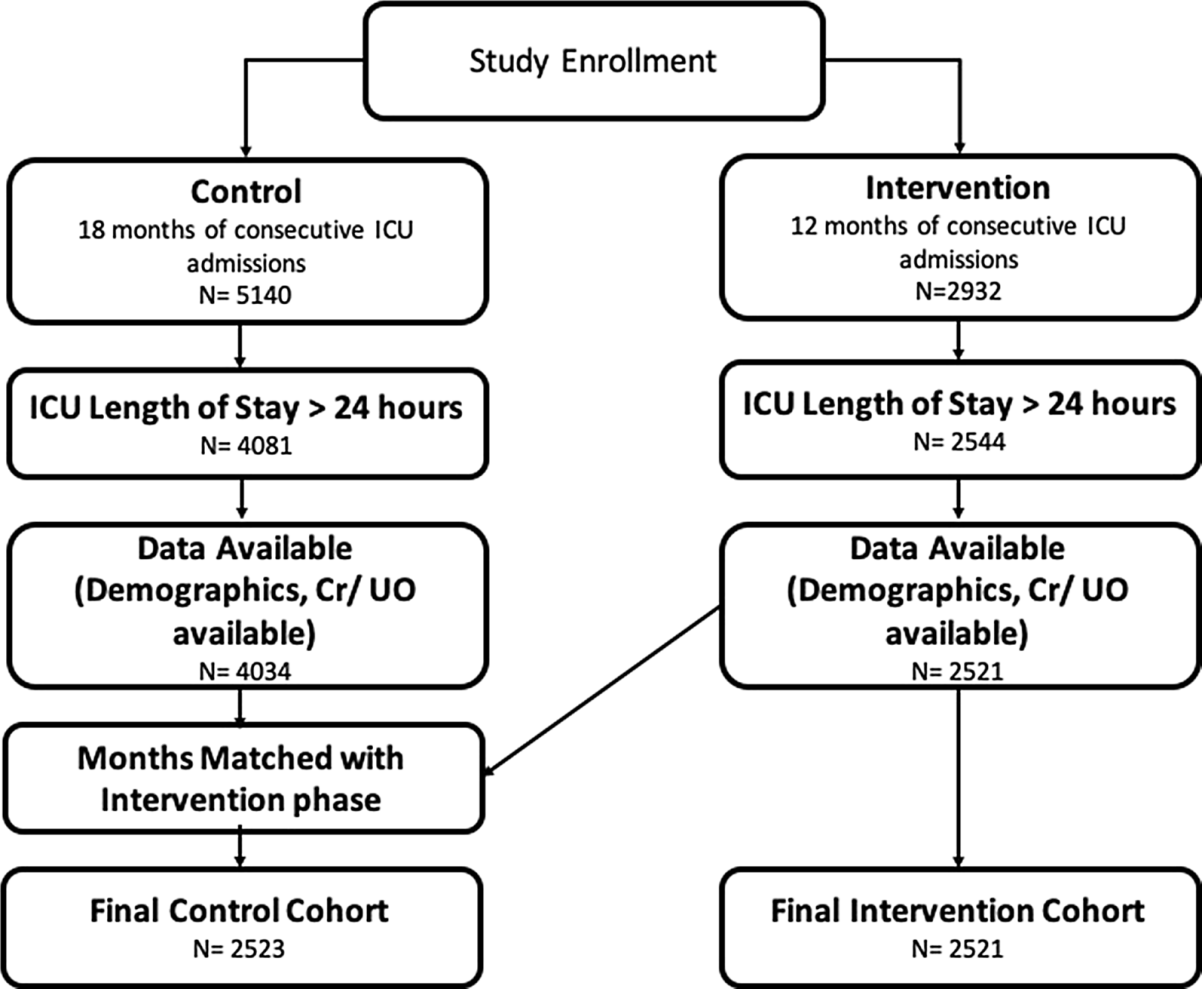
Patient enrollment and selection for analysis shown for the control and intervention cohorts. Numbers in each box indicate number of admissions remaining after applying each inclusion criteria. The control cohort is matched in time to the intervention cohort. See text for more details

The study was divided into two phases—the control phase when no AKI guidelines and care bundle was shown to clinicians and the intervention phase when AKI guidelines and care bundle was available to clinicians via ICCA. The control phase consisted of retrospective data from consecutive adult patients admitted to GICU and CICU over 18 months for more than 24 h. The intervention phase consisted of adult admissions to the same units over 12 months for more than 24 h. For both phases, encounters were excluded from analysis if (1) age, race or gender information was not available, (2) age at admission > 90 years, (3) had acute kidney injury on admission defined as oligoanuria in the first 6 h of admission, (4) patients received renal replacement therapy (RRT) during ICU stay. (Patients receiving RRT were excluded from the analysis of AKI scores due to confounding of the urine output and creatinine values by the RRT, giving a false score output from the algorithm. The use of RRT was displayed as AKI Stage 3 in the decision support tool). Additionally, encounters where insufficient data were available to calculate AKI score (urine output, body weight, serum creatinine) were excluded. The institutional review authority deemed this investigation a quality improvement project and waived the requirement for patient consent. Figure 1 shows the patient selection process for the control and intervention phase.

### Intervention design and implementation

The intervention consisted of three components—(1) automated AKI guidelines, (2) AKI care bundle tailored to workflow and (3) education and training about AKI guidelines and the care bundle.

The automated electronic AKI staging guidelines were based on AKIN [13]. The automated electronic AKI staging algorithm has been previously developed and validated [12]. The AKI stage is determined to be the maximum of the most recent values of AKI using urine output staging (AKI UO) and AKI using serum creatinine staging (AKI Cr). AKI UO is calculated as described in the AKIN guidelines using weight normalized hourly urine output calculated over different time periods for different stages. AKI Cr is also calculated per guidelines, based on the increase in measured serum creatinine from baseline serum creatinine. This algorithm was integrated into ICCA (version H.02.01). AKI Cr and AKI UO each were calculated every time; a new measurement of serum creatinine and a new measurement of urine output, respectively, were charted in ICCA.

The AKI care bundle was implemented in ICCA and comprised of five following elements;

1. AKI flowsheet displaying:
  a AKI score
  b Urine output
  c Creatinine
  d Blood gases
  e Medications
  f Fluid input/output
2. Two advisories which require acknowledgment and prompt intervention by the clinical team:
  a AKI Deterioration advisory
  b Enoxaparin dose alert
3. AKI care bundle form displaying:
  a AKI stage
  b Current nephrotoxic medications
  c List assessments and interventions to consider
4. AKI order set which the physician can use to ensure compliance with AKI guidelines. Suggested actions include
  a Measure serum creatinine more frequently
  b Stop nephrotoxic drugs.
5. An AKI dashboard which provides patient overview for the whole unit was placed in the corridor of the unit for easy access. It displayed
  a The AKI score
  b Change in AKI score
  c Recommendations for enoxaparin dosing

**Fig. 2:**
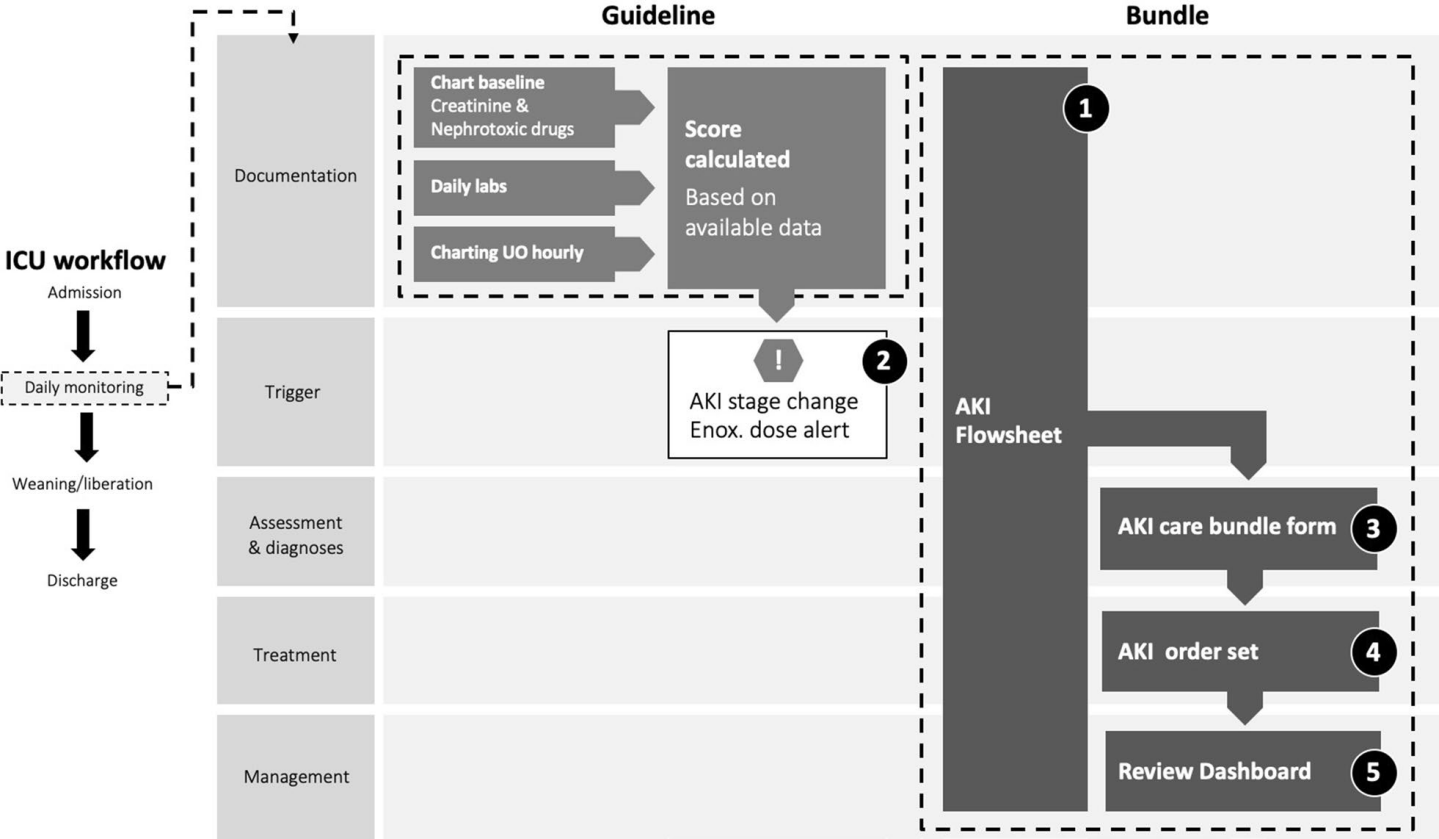
Schematic representation of the study protocol. Two components of the intervention-guideline and bundle are shown mapped to aspects of clinical workflow (left column) which they affect. Each component of the intervention is numbered corresponding to the description in Methods. See ‘Methods’ section for details

These five elements are mapped to elements of the workflow in Fig. 2. The first four elements of the care bundle were implemented in both units. The AKI flow sheet and advisories are visible to all members of the multidisciplinary team (MDT) (doctors, nurses and pharmacists). Advisories can be acknowledged but no aspect of the decision support tool is mandatory. The AKI bundle form and AKI order set are available to doctors. In GICU, the dashboard is visible to all members of the MDT. The dashboard was not implemented in CICU due to lack of resources. In addition, the admission document for both units was modified to include charting of baseline serum creatinine and listing nephrotoxic medications currently prescribed according to a predefined list.

The third component of the intervention was training and education for the clinicians in the two units. To facilitate this, a video was created to explain how AKI scores are calculated and how to use the care bundle. This video was shared with the clinicians and made accessible in both units. There was no additional teaching on AKI during the study but rotating junior doctors in GICU all received instruction on AKI management as part of their scheduled teaching program. Nursing staff had no additional education on AKI management.

There were no other changes in clinical practice during the study, DVT prophylaxis protocols were the same, and there were no additional staff including specialist AKI nurses or additional pharmacists. The use of the new workflow was highlighted as part of daily safety briefs during morning rounds.

### Data extraction and AKI computation

Data were extracted from CCIS to compute the AKI score and evaluate the outcomes. All data were anonymized to remove identifiable information and all timestamps were shifted. Extracted data included demographic information such as age, gender, ethnicity, ICU admission and discharge time; serum creatinine measurements, baseline serum creatinine at admission and urine output for calculating the AKI stage. Enoxaparin doses and estimated glomerular filtration rate (eGFR) for evaluating enoxaparin outcomes were also extracted.

Continuous AKI scores were calculated for the control phase using electronic sniffer algorithm and have been previously described in a previous publication [14]. AKI scores were calculated anytime a new urine output or a new serum creatinine measurement was charted. To calculate AKI Cr, a baseline serum creatinine value is needed. In the control phase, this value was mostly not available; therefore, the MDRD equation [15] was used to estimate baseline creatinine for the entire cohort. In the intervention phase, majority of the patients had baseline serum creatinine charted. For the rest of the patients, MDRD equation was used to calculate baseline creatinine values. To calculate AKI UO, urine output values were normalized by the most recently measured weight (daily or admission weight).

### Outcomes evaluation

The primary outcome of the study was the proportion AKI patients developing a worse stage of AKI during their stay. Additionally, the proportion of correctly administered enoxaparin doses was used as a measure of clinician awareness of AKI & guideline compliance. The secondary outcomes are shown in Table 1. The time-series values of AKI (AKI UO and AKI Cr), eGFR and administered enoxaparin dose were used to compute these outcomes. The outcomes were then compared between the control phase and the intervention phase. The analysis further done for each ICU unit separately and results are reported.

**Table 1:** Secondary outcomes and definition

Outcome	Definition
AKI at admission	The first AKI stage computed within 6 h of ICU admission was used as the AKI stage at admission since creatinine and urine output measurements prior to ICU admission were unavailable. If this value was 1 or greater, the encounter was labeled as having AKI at ICU admission
AKI at discharge	The last measured value of urine output or serum creatinine was used to calculate AKI stage and this value was used as the AKI stage at discharge
Maximum AKI	The maximum value of AKI stage during each encounter was calculated and used to assess AKI score distribution
Maximum AKI per day	The maximum AKI stage per 24-h period in an ICU stay was calculated and used to estimate the prevalence of AKI in each ICU day. This was done for the first 5 days of ICU admission. Encounters with AKI at admission (as defined above) were excluded from this analysis

The table lists secondary outcomes for the study and their definition. Results of these outcomes are described in ‘Results’ section

To determine the primary outcome, i.e., increasing AKI risk from Stage 1, the number of encounters where there was at least one increase from Stage 1 to a higher AKI stage, was calculated. In addition, the number of encounters where there was at least one increase from Stage 2 to Stage 3, was also calculated.

To evaluate guideline compliance, the correct dose was defined as an amount equal to or less than the recommended daily enoxaparin dose based on renal function (See Additional File 1: Table S1). To evaluate if the correct dose was given, we extracted the dose and timing information for all nonzero doses of enoxaparin. The closest preceding eGFR value was also extracted. Every enoxaparin dose was classified as correct or incorrect based on the preceding eGFR value. The total number of correct and incorrect doses per encounter and for each phase of the study was calculated. Any enoxaparin dose given before eGFR was available was removed from the analysis. Patients who received dialysis or weighed more than 100 kg were also excluded from this analysis. In addition to evaluating by dose, we also computed the number of admissions in which correct dose of enoxaparin was given for the entire ICU stay.

### Statistical measures and powering study

The primary outcome was evaluated after control phase data were extracted and have been published previously [14]. The primary outcome-rate of AKI stage increase was used to power the study. The proportion AKI patients progressing to a worse stage of AKI was used to determine the study sample size. To show a reduction in 15% of the number of patients progressing to a worse AKI stage, a study size of 6000 admissions (3000 controls and 3000 main phase) is needed for power of 0.86 and α error of 0.04 (using Fisher’s exact one-tailed test). Based on available information on the number of patients per year, it was estimated that 3000 patients could be recruited in 12 months. This includes a portion of cohort which might be excluded from analysis due to insufficient data or not meeting inclusion criteria.

The intervention phase of the study was conducted for 12 months. From the 18 months of data in the control phase, 12 months were selected to time-match with months of the intervention phase. This accounted for the seasonal variations in workforce and patient population. The statistical significance of the primary and secondary outcomes was evaluated using binomial proportions test implemented in Python 3.7 Scipy (version 1.3.1). Student’s *T* test was used to evaluate statistical differences between cohort demographics.

Kaplan–Meier survival curves were generated using time to AKI as the event of interest. Patients who were discharged or developed AKI each day were removed from analysis to generate these curves. This analysis was done for both phases.

## Results

### Demographics

In control phase, out of 5140 consecutive ICU admissions over 18 months, 2523 ICU admissions were selected for data analysis and comparison. In the intervention phase, 2932 consecutive ICU admissions were enrolled into the study. After applying the exclusion criteria, 2521 ICU admissions were used for analysis (Fig. 1). Table 2 shows the patient cohort demographics in the two phases of the study. The two cohorts were similar in age, ICU length of stay, gender distribution and distribution in the two units. Admission weight was significantly higher in the intervention phase and in the intervention phase the proportion of black patients was significantly higher, although a very small proportion of the population. The proportion of patients getting RRT in ICU was significantly higher in the intervention phase (7.9% in intervention phase vs 6.5% in control phase), the proportion of readmissions was significantly higher in the intervention phase.

**Table 2:** Cohort overview

	Control	Intervention	*p* value
Patients	2389	2394	
Encounters	2523	2521	
Gender	811 F (32.1%)	813 F (32.2%)	0.94
Clinical unit (GICU)	1055 (41.8%)	1120 (44.4%)	0.06
Age	63.6 (14.5) years	63.0 (15.1) years	0.15
ICU LOS	5.8 (6.7) days	5.9 (6.0) days	0.58
RRT	163 (6.5%)	200 (7.9%)	*0.04*
Readmissions	46 (1.8%)	81 (3.2%)	*0.002*
Weight (kg)	81.0 (20.0)	84.7 (20.9)	< *0.001*
Race (Black)	33 (1.3%)	81 (3.2%)	< *0.001*
Baseline creatinine measured	41 (1.63%)	1463 (58.03%)	< *0.001*
Encounters with SCr measurements	2523 (100%)	2516 (99.8%)	*0.025*
Encounters with UO measurements	2437 (96.6%)	2426 (96.2%)	0.49
Encounters with weight measurements	2406 (95.4%)	1829 (72.6%)	< *0.001*

The table compares the control and intervention cohort demographics and data availability. Data are presented as: for binary variables—count (percentage of admissions) and for continuous variables—mean (standard deviation). Details of *p* value calculation are provided in Methods and statistically significant differences are highlighted in italics font. See ‘Results’ section for details

Entering baseline serum creatinine value was a part of the intervention. The proportion of admissions with a value increased from 1.6 to 58% in the intervention phase.

In the addition to these demographic features, we also compared demographic, admission and discharge features in each unit (Table 3). Hospital length of stay was significantly longer in the intervention phase in both units. ICU length of stay was similar in the GICU in both phases. In CICU, the ICU length of stay was longer by 0.4 days in intervention phase, which was statistically significant. The proportion of medical and surgical admissions was similar in the two phases in GICU; however, the nature of surgery was statistically different. In GICU, the proportion of emergency/urgent surgery was significantly higher, while the proportion of urgent and elective/scheduled surgery was significantly lower in the intervention phase. In CICU, the proportion of urgent surgeries was significantly lower, while the proportion of elective/scheduled surgeries was significantly higher in intervention phase. Among outcomes, mortality was significantly lower and readmissions significantly higher in CICU. No significant difference was observed in the GICU.

**Table 3:** Unit-wise admission and outcomes

	General ICU			Cardiac ICU		
	Control (*n* = 1041)	Intervention (*n* = 1022)	*p* value	Control (*n* = 1346)	Intervention (*n* = 1363)	*p* value
Age	61.0 (15.6)	60.2 (16.5)	0.26	65.4 (13.3)	65.3 (13.4)	0.72
ICU LOS	6.1 (8.0)	5.7 (7.4)	0.22	5.6 (5.7)	6.0 (4.6)	0.04
Hospital LOS	15.5 (18.6)	17.4 (19.2)	0.02	11.4 (9.1)	13.0 (9.8)	< 0.0001
*Admission information*						
Medical	546 (52.4%)	572 (56.0%)	0.11	0 (0%)	0 (0%)	NA
Surgery	495 (47.6%)	450 (44.0%)	0.11	1346 (100%)	1363 (100%)	NA
Emergency/salvage	45 (4.3%)	94 (9.2%)	< 0.001	55 (4.1%)	58 (4.3%)	0.83
Urgent	110 (10.6%)	73 (7.1%)	0.006	502 (37.3%)	300 (22.0%)	< 0.001
Elective/scheduled	340 (32.7%)	283 (27.7%)	0.014	789 (58.6%)	857 (62.9%)	0.023
*Outcomes*						
APACHE II/EuroScore (CICU control)	15.3 (7.1)	15.0 (6.6)	0.33	5.3 (2.9)	8.9 (8.2)	NA
APACHE II mortality prediction	19.6 (21.6)	19.1 (19.6)	0.58		4.9 (8.0)	NA
Readmissions	16 (1.5%)	21 (2.1%)	0.38	30 (2.2%)	60 (4.4%)	0.002
Died	132 (12.7%)	107 (10.5%)	0.12	29 (2.16%)	12 (0.88%)	0.007

The table shows ICU and hospital stay information, admission information including type of surgery and outcomes for each unit separately. Data are presented as: for binary variables—count (percentage of admissions) and for continuous variables—mean (standard deviation). *p* values compare control cohort to intervention cohort for each unit. *p* value calculation is described in ‘Methods’ section. See ‘Results’ section for details

### Primary objective: proportion of patients deteriorating from Stage 1 AKI

**Fig. 3:**
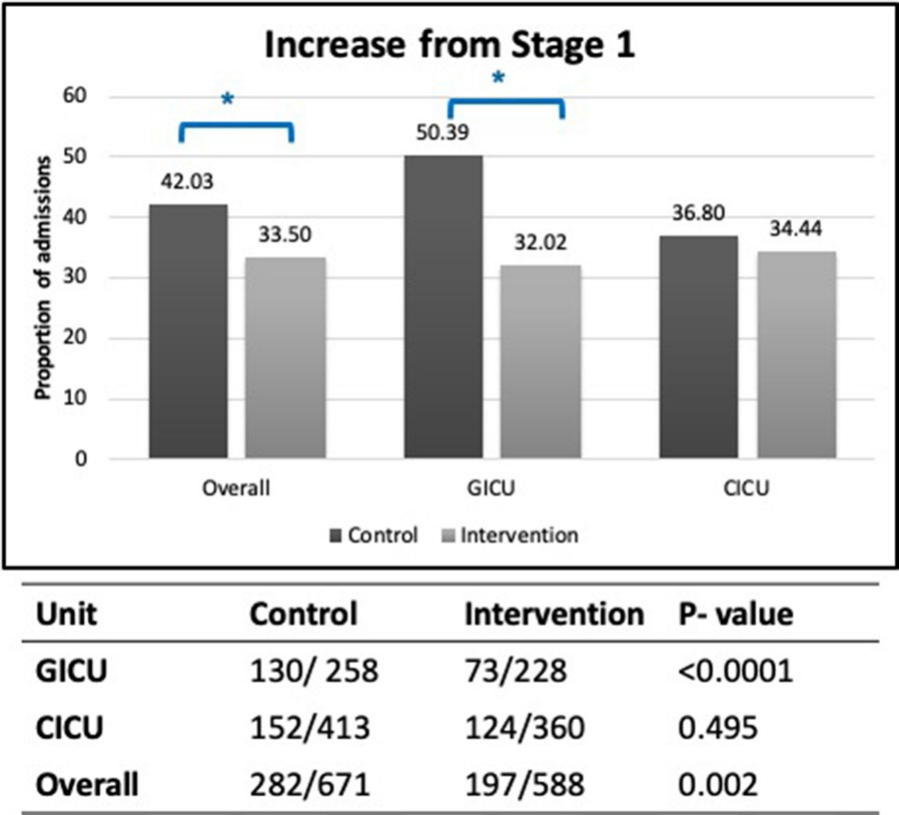
Primary outcome-proportion of patients deteriorating from Stage 1. Top: Graph of percentage of admissions with AKI Stage 1 worsening to higher AKI Stage for the overall cohort and GICU and CICU separately (control = dark gray; intervention = light gray). Number on each bar is actual percentage. Asterisk denotes statistically significant difference. Bottom: Number of admissions who worsen to higher stage of AKI (from Stage 1)/total number of admissions for each cohort and unit type. See text for details

The proportion of Stage 1 AKI patients progressing to higher AKI stages, decreased in the intervention phase (42–33.5%, *p* = 0.002) (Fig. 3). In the GICU, the proportion of deteriorations decreased from 50.4% in control phase to 32% in intervention phase (*p* < 0.001), while in CICU the proportions decreased from 36.8% in control to 34.4% in intervention (*p* = 0.5). The proportion of encounters in Stage 2 increasing risk to Stage 3, decreased from 21.3% in control phase to 11.8% in intervention phase (*p* = 0.005).

### Guideline compliance metric: enoxaparin dose compliance

**Fig. 4:**
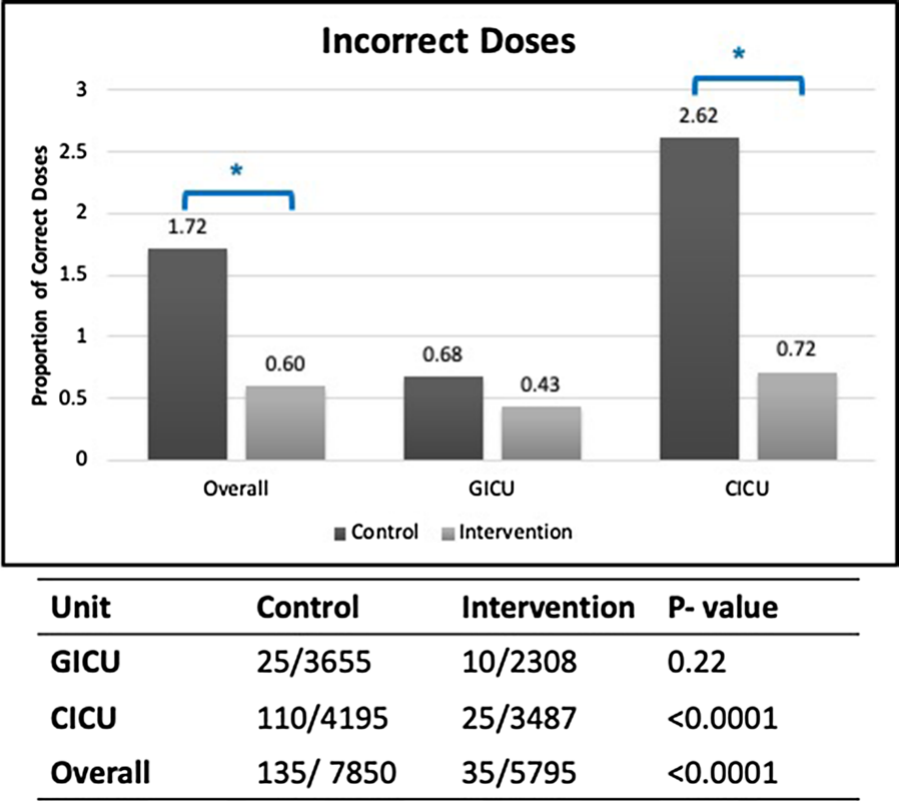
Guideline compliance-proportion of incorrect enoxaparin doses. Top: Graph of proportion of correct doses as a fraction of total doses for the overall cohort and GICU and CICU separately (control = dark gray; intervention = light gray). Number on each bar is actual percentage. Asterisk denotes statistically significant difference. Bottom: Number of incorrect doses/total number of doses for each cohort and unit type. See text for details

The adherence to eGFR-based enoxaparin dosing guidelines was evaluated as described above and see Additional file 1: Table S1. The proportion of incorrect doses decreased from 1.72% in control phase to 0.6% in the intervention phase (*p* < 0.001). This decrease was largely from the CICU where the proportion of incorrect doses decreased from 2.62% in control phase to 0.72% in the intervention phase. The GICU, in contrast had lower proportion of incorrect doses, which decreased from 0.68 to 0.43% (Fig. 4).

The analysis of wrong doses grouped by eGFR value and dose revealed that the majority of the incorrect dosing occurred in eGFR range 20–30 ml/min/1.73 m^2^ when 40 mg dose of enoxaparin was given (correct dose is 20 mg). The second frequent wrong dosing occurred in the eGFR range < 20 ml/min/1.73 m^2^ when 40 mg of enoxaparin was given (correct dose is 0). For both these cases and other cases, the number of wrong doses decreased in both units in intervention phase.

Evaluating guidelines compliance by encounter, a statistically significant increase in the number of encounters who received correct dose for their entire ICU stay, was observed in the intervention phase (control phase = 29.8%, intervention phase = 52.1%; *p* = 0.011). This increase was largely driven by the CICU where the proportion of increased from 21.4 to 53.3%. The GICU had a smaller increase from 46.4 to 50%.

### Secondary objectives

#### AKI at admission and discharge

The proportion of patients admitted with AKI was similar in the two phases, but the proportion of patients discharged with AKI was significantly lower in the intervention phase (Table 4 and Additional file 1: Fig. S1). The proportion of patients admitted with no AKI and developing any AKI stage in ICU decreased significantly from 33.9% in the control phase to 29% in the intervention phase (*p* < 0.001). In each ICU, there was a decrease in the proportion of patients developing AKI, but this was statistically significant only in the GICU.

**Table 4:** AKI statistics

Metric	Control (*n* = 2523)	Intervention (*n* = 2521)	*p* value
Admitted with AKI	163	138	0.14
Developed AKI in ICU	855	732	*0.00021*
Discharged with AKI	242	188	*0.0066*
Max AKI Stage 1	671	588	*0.0073*
Max AKI Stage 2	272	229	*0.044*
Max AKI Stage 3	75	53	*0.049*

The table shows AKI statistics for control and intervention cohort with *p* values. The first three rows are the number of admissions with AKI at admission and discharge. The last three rows show the number of admissions with maximum AKI during ICU stay with that AKI stage. Statistically significant *p* values are indicated in italics. See ‘Results’ section for more details

#### AKI prevalence (maximum AKI during ICU stay)

The proportion of patients with any AKI stage was significantly lower in the intervention phase (Table 4 and Additional file 1: Fig. S2). The proportion of patients with maximum of AKI Stage 1 decreased from 28.4 to 25.3%, AKI Stage 2 decreased from 11.5 to 9.9% and AKI Stage 3 decreased from 3.2 to 2.3%. Conversely, the proportion of patients who didn’t develop AKI was significantly higher in the main phase (56.9% in control phase vs. 62.5% in intervention phase, *p* < 0.01). This trend was observed in both ICUs with the proportion of patients with any stage of AKI decreased in the intervention phase in both units.

#### Maximum AKI per day

The distribution of daily maximum AKI stages for first 5 days of ICU stay is shown in Additional File 1: Fig. S3. Overall, the proportion of patients with AKI increased from Day 1 to Day 3 of ICU stay and decreased thereafter. The relative distribution of the three AKI stages is similar between the control and intervention phase. However, the proportion of patients without AKI each day is higher and the proportion of patients in each stage of AKI each day is lower in the intervention phase.

### Kaplan–Meier analysis

**Fig. 5:**
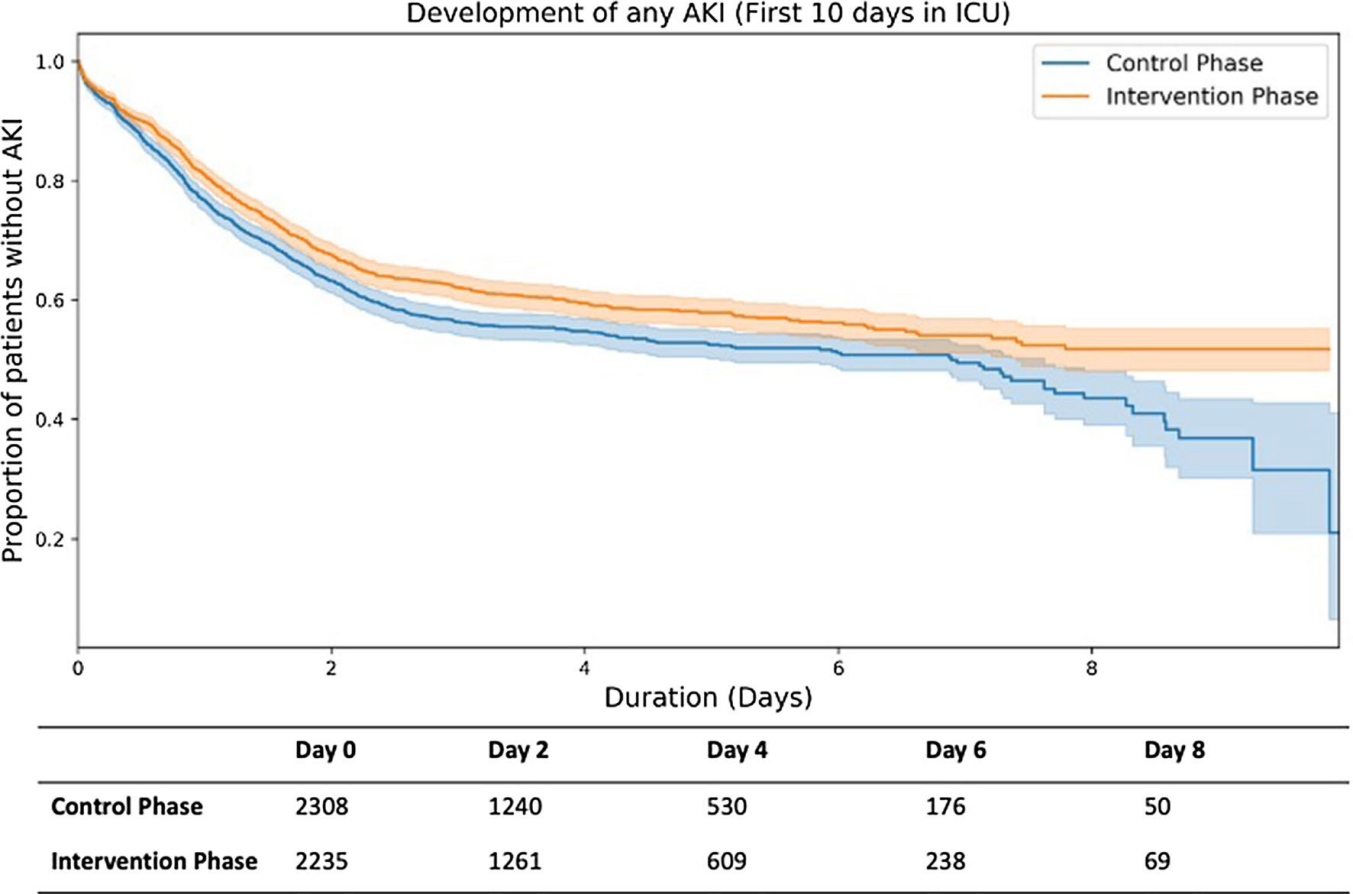
Kaplan–Meier Curve for AKI development. Top: Trendline of proportion of patients without AKI as a function of duration (ICU days). The orange line shows the intervention phase and blue line shows the control phase. Shaded regions show 95% confidence interval. Bottom: Number of admissions without AKI for the given ICU day in the control and intervention cohort

The Kaplan–Meier curves plotting the cumulative proportion of patients with no AKI over days in ICU are shown in Fig. 5. These curves are shown for the first 10 days of ICU stay for the control and intervention phase. The cumulative proportion curve for the intervention phase is higher than the control phase indicating that in each successive day fewer patients developed AKI in the intervention phase. The table below shows the number of patients with no AKI at different ICU stay days.

## Discussion

This study evaluated the impact of a complex intervention including a clinical decision support system (CDSS) for the early detection and management of AKI, in two separate critical care units at a large UK hospital. It was a before and after design utilizing routinely collected data from 2 matched 12-month periods.

Most studies evaluating the implementation of electronic alerts, like ours, are limited by the before and after design. Several of these studies have demonstrated an association with reduced time to therapeutic intervention [8], more rapid return to baseline renal function, reduced length of hospital stay [16, 17] and reduced mortality [18, 19]. However, the results were less promising in other studies. Wilson et al. [7], for example, did not find an improvement in AKI care following introduction of a single AKI alert in a randomized controlled trial in a whole hospital setting. There was also no difference shown for the subset of sicker patients in the intensive care unit. Using a stepped wedge cluster randomized study design, Selby et al. [9] did not find an improvement in mortality but did find evidence of improved recognition of AKI as well as reduced hospital length of stay. The variability in results indicates the importance of combining clinical decision support tools with effective implementation strategies.

Proactive drug dosing and avoidance of nephrotoxic agents by clinicians are vital elements in the management of AKI. The results of studies examining the impact of CDSS in AKI vary. Improved prescribing practices for patients with acute kidney injury were demonstrated by Field et al. [20] where final drug orders were appropriate significantly more often when an electronic alert was provided for patients with renal impairment in a long-term care facility. Terrel et al. [21] showed a reduction in excessive drug dosing in the emergency department (ED) with a CDSS based on creatinine; however, a web-based surveillance tool implemented in a study by Mccoy et al. [22] did not reduce the time to alteration of renally cleared or discontinuation of nephrotoxic medication. A screening tool used to identify pediatric patients exposed to multiple or prolonged courses of nephrotoxic agents was effective in reducing exposure to nephrotoxic drugs and the occurrence of AKI overall.

Our results demonstrate a reduction in the progression of AKI from Stage 1 to higher stages after implementation of the CDSS. These results are consistent across both units. We also found a reduction in number of patients progressing from Stage 2 to Stage 3 AKI. This work also demonstrated improved compliance with enoxaparin prescribing guidelines. DVT prophylaxis is an important component of standard ICU care and enoxaparin is a commonly used first-line agent for prophylaxis. While the dose should be reduced in AKI, dose adjustment is commonly overlooked by the clinical team and therefore could act as a surrogate marker of recognition of AKI. Our study showed a significant improvement in enoxaparin dose adjustment. In the control phase, we identified that most enoxaparin dosing errors occurred in patients with eGFR between 20 and 30 ml/min/1.73 m^2^ (1.4% of all enoxaparin doses). In the intervention phase, we observed this dosing error was significantly lower (0.6% of all enoxaparin doses, *p* < 0.001). In addition, in patients with eGFR < 20 ml/min/1.73 m^2^, dosing error was similarly reduced.

We also demonstrated a significant reduction in the overall incidence of AKI within our patient population (Table 4) including a reduction in patients developing any AKI risk during ICU stay and a reduction in proportion of patients in every AKI stage. The analysis of AKI progression over ICU stay (Fig. 5 and Additional file 1: Fig. S3) shows the reduction in the number of patients in any AKI stage can be seen from ICU admission and is sustained throughout the ICU stay. Since the alert and order set only appeared following the development of Stage 1 AKI, this would not be attributable to the CDSS itself but may be explained by an increased staff awareness of AKI due to education, awareness of the project, appearance of alerts on other patients and importance of certain interventions in preventing AKI.

The intervention also included charting baseline creatinine at admission, which increased from 1.6 to 58%. The availability of charted baseline creatinine improved staff awareness and may have contributed identification of community-acquired AKI, risk stratification of patients at risk of developing AKI, ultimately leading to decrease in AKI prevalence in the intervention phase. An additional benefit of charting baseline creatinine at admission is improved accuracy of AKI Cr staging (when compared to empirical estimates using MDRD equation).

Our study was conducted in 2 separate ICU’s each with significant differences in their patient populations and medical staffing models. CICU admits elective postoperative cardiac surgical patients and has fewer consultant and junior medical staff. The GICU admits a broader population of emergency and elective medical and surgical patients. We reported in previous work that the pattern of AKI development in these populations is different [14]. It is likely that the precipitants of AKI are disparate between the 2 groups and therefore opportunities and strategies to intervene in AKI may vary. For CICU patients the precipitating event occurs at the time of surgery and shortly afterward. Renal function is often normal at admission and deteriorates in the first 24–28 h postoperatively.[23]. For the GICU population, the insult is often progressive, starts prior to admission and continues until the reversal of the underlying cause [24]. Our results indicate that while the progression of AKI may be different in the different populations, both populations benefitted from the applied care bundle.

Kashani [25] highlighted important considerations when developing an effective electronic alert which we incorporated into our design combined with training and clear clinical instructions to encourage behavioral change. These included identification of high-risk patients, targeting the alert directly to the clinical team together with advice to implement an order set designed to avert further renal insult. This was followed by real-time display of AKI stage for individual patients (graphically and numerically) on a centrally located dashboard. One criticism of the intervention was that once an alert was triggered the CDSS was not launched automatically, instead requiring a clinician to specifically implement it on the CCIS. These requests were not uniformly completed. One could speculate that once alerted and with a simple, specific and easily remembered care bundle, the relevant actions were carried independently by responsible clinicians without the requirement for launching a specific ‘aide memoir’ within the system. Another limitation of this study is that it was designed as a before–after study and designed to be correlational. Therefore, we are unable to attribute the improvements in patient outcomes to a specific aspect of the intervention.

## Conclusions

This large observational study demonstrated significant improvement in AKI progression from Stage 1 to higher stages and improved compliance with enoxaparin prescribing guidelines in the context of AKI. The overall incidence of AKI was also reduced. The study utilized automated alerts and CDSS within a CCIS. To date, it is the largest study of such an intervention in the critical care population. We found that a number of enhancements to the CDSS may further enhance the impact and we recommend a larger, more robust trial to examine the potential benefit of this system.

## Supplementary information

**Additional file 1.** Additional file containing table and figures providing additional information as referenced in the text.

## Data Availability

The data that support the findings of this study are available from University Hospitals Bristol NHS Foundation Trust but restrictions apply to the availability of these data, which were used under license for the current study, and so are not publicly available. Data are, however, available from the authors upon reasonable request and with permission of University Hospitals Bristol NHS Foundation Trust.

## Abbreviations

AKI: Acute kidney injury
AKI UO: AKI urine output
AKI Cr: AKI serum creatinine
CDSS: Clinical decision support system
CISS: Critical care informatics system
ICU: Intensive care unit
CICU: Cardiac ICU
GICU: General ICU
ICCA: IntelliSpace Critical Care and Anesthesia
eGFR: Estimated glomerular filtration rate
MDRD: Modification of diet in renal disease
RRT: Renal replacement therapy
ED: Emergency department
DVT: Deep vein thrombosis
