# Supplementary material for: Impact of a computerized decision support tool deployed in two intensive care units on acute kidney injury progression and guideline compliance: a prospective observational study
Source: Crit Care. 2020 Nov 23;24:656. doi: 10.1186/s13054-020-03343-1 (PMC7684927; doi:10.1186/s13054-020-03343-1)

Additional file Table and Figures

Table of Contents

Additional file Table S1 2

Additional file Figure S1 3

Additional file Figure S2 4

Additional file Figure S3 5

# Table S1

Recommended enoxaparin dose for AKI Stages and eGFR value

| **Recommended Enoxaparin Dose** | **eGFR value** | **AKI Stage** |
| --- | --- | --- |
| 40 mg/ day | >30 | Stage 0 |
| 20 mg/ day | 20- 30 | Stage 1 & Stage 2 |
| 0 | <20 | Stage 3 |

# Figure S1


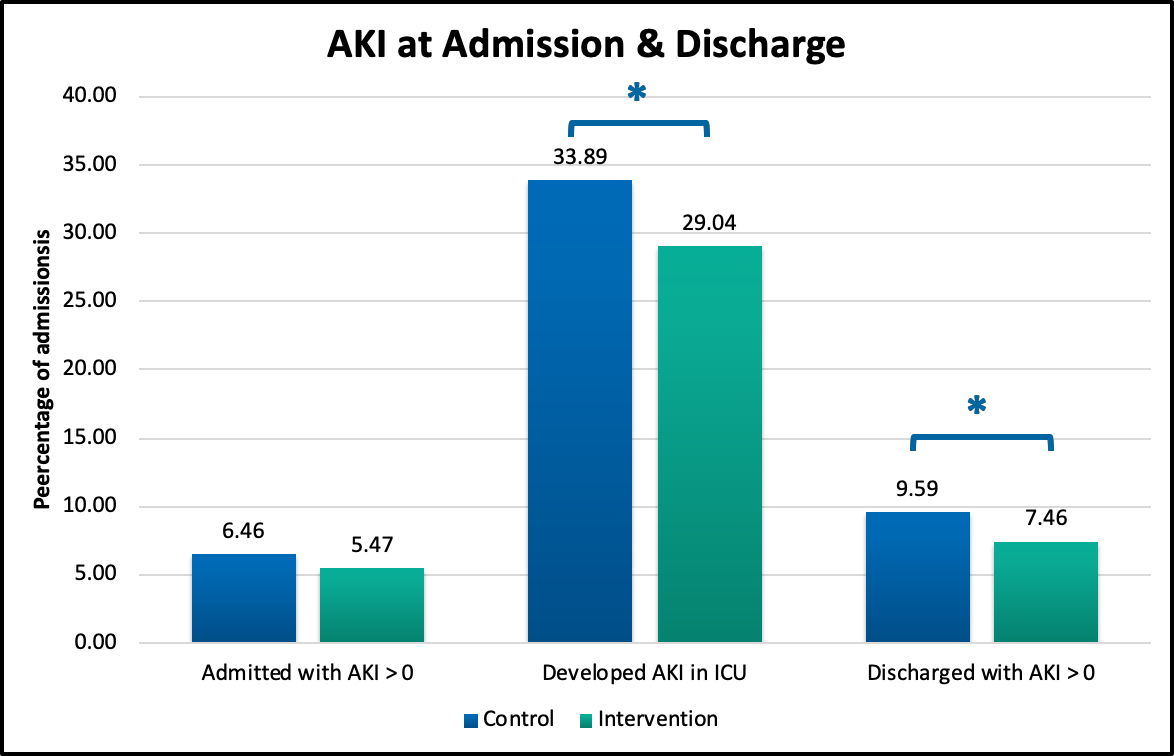
Proportion of admissions with AKI at admission (left), admitted without AKI and developed AKI in ICU (middle) and AKI at discharge (right). Asterisk indicates statistically difference. Numbers on each bar are the percentage of admissions in each category. See text for details.

# Figure S2

Proportion of admissions with given maximum AKI stage during ICU stay for each cohort (control= blue; intervention= green). Asterisk indicates statistically significant difference. Numbers on each bar shows the percentage of cohort. See text for details.


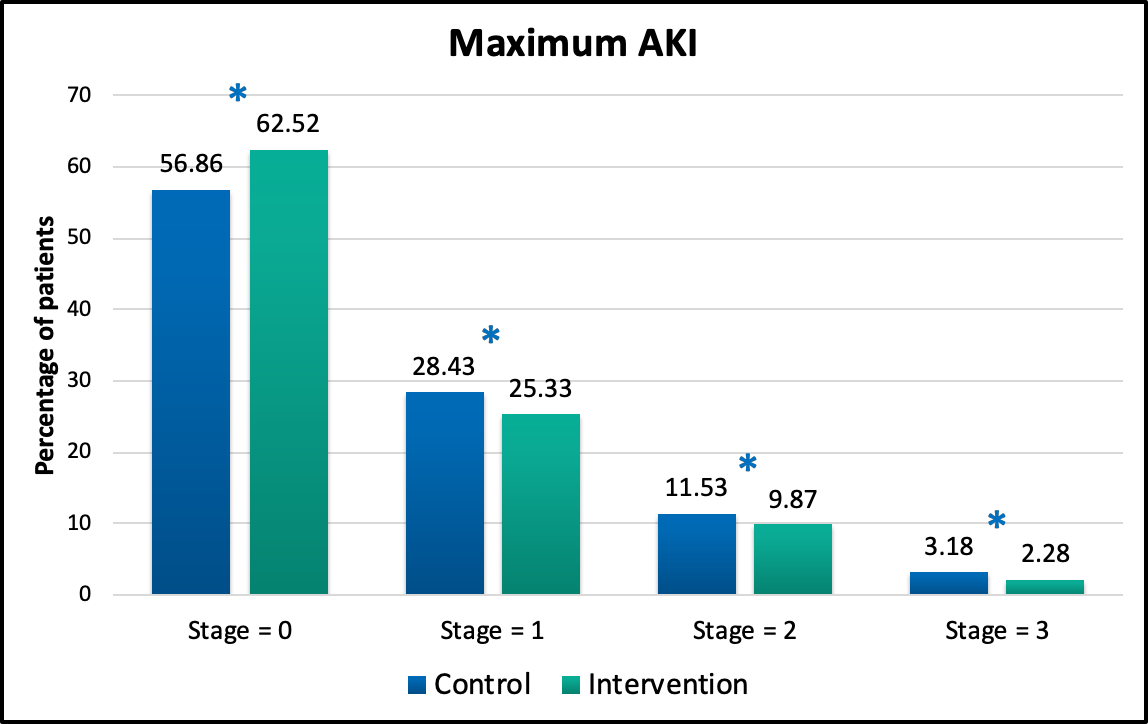


# Figure S3

AKI stage distribution for control (left) and intervention (right). The graphs show the distribution of admissions with given maximum AKI stage per ICU day for first 5 days of ICU stay. Colors represent the different AKI stage. Number of patients in each AKI stage per day are shown in each section. See text for details.


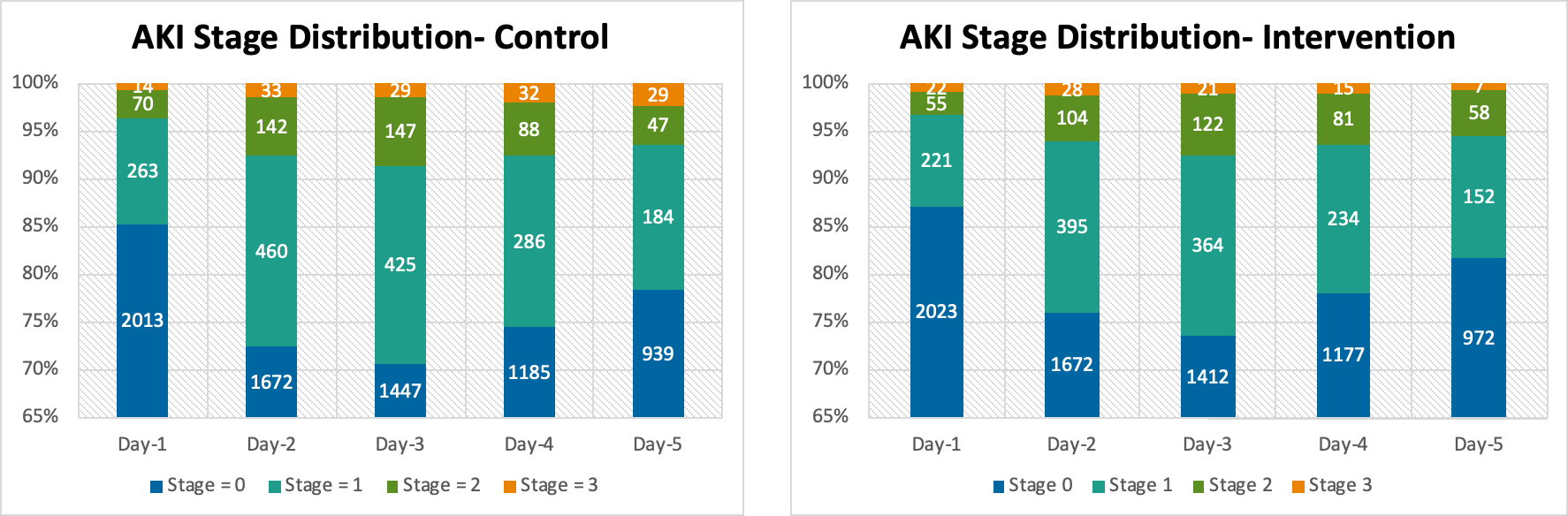

Supplement: Supplementary file 1 — Additional file 1. Additional file containing table and figures providing additional information as referenced in the text. [file 13054_2020_3343_MOESM1_ESM.docx]
